# Reversible control of cell membrane receptor function using DNA nano-spring multivalent ligands†
†Electronic supplementary information (ESI) available: Sequence information and synthesis of the RGD–DNA conjugate. See DOI: 10.1039/c7sc02489d



**DOI:** 10.1039/c7sc02489d

**Published:** 2017-08-18

**Authors:** Kaixiang Zhang, Ruijie Deng, Yupeng Sun, Ling Zhang, Jinghong Li

**Affiliations:** a Department of Chemistry , Key Laboratory of Bioorganic Phosphorus Chemistry & Chemical Biology , Tsinghua University , Beijing 100084 , China . Email: jhli@mail.tsinghua.edu.cn

## Abstract

Chemically functionalized and nanostructured materials, which mimic the features of the natural extracellular matrix, provide a tool to organize cell surface receptors into nanoscale clusters and manipulate cell functions. However, the existing materials are mainly based on static structures. Herein, we developed a DNA based structure-switchable and multivalent material that acts as a ‘nano-spring’, enabling reversible control of membrane receptor function at the cell surface. This ‘nano-spring’ can be easily synthesized by rolling circle amplification and finely tuned by changing the circular template design. Using this ‘nano-spring’ to interact with cells, we have demonstrated that the movement of the DNA nanostructure is sufficient to direct a cell morphology change from the normal morphology to having numerous cell protrusions and affect the mRNA expression level of integrin related genes. This DNA nano-spring structure can be a competitive material for actively manipulating cell receptor function and may help us to understand the role of receptor mediated signalling cascades.

## Abstract 2

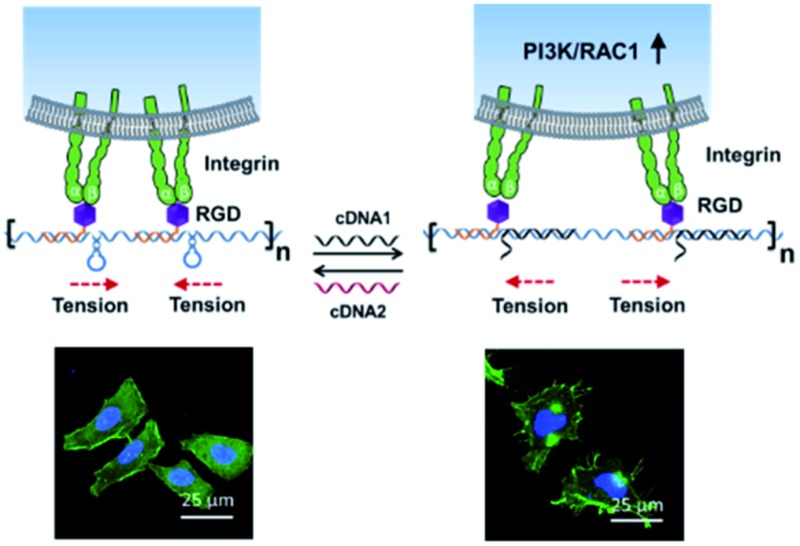
DNA nano-spring multivalent ligands for reversibly controlling the nanoscale distribution of cell binding ligands and regulating the cell behavior.

## Introduction

Cells constantly sense extracellular environmental cues with surface receptors and adjust their intracellular functions to respond.^1–4^ Interestingly, many receptors do not operate as individual entities, but rather collaborate as a complex.^5–7^ Mounting evidence has suggested that integrin receptor activation (essential for cell spreading, proliferation and migration^8^) is highly dependent on their nanoscale arrangement.^9–12^ Therefore, controlling the spatial distribution of cell surface receptors is important both for the study of fundamental signalling pathways and for the external control of cell behavior.

Synthetic extracellular matrices with nanoscale features have been utilized to organize cell surface receptors into clusters, control cell fate and illustrate underlying cell function mechanisms.^13–16^ For example, Schaffer’s group demonstrated that hyaluronic acid polymer based multivalent conjugates could organize neural stem cell receptors (ephrin-B2) into clusters and control stem cell behavior.^17^ Mooney and colleagues utilized a synthetic alginate extracellular matrix to illustrate that the clustering of integrins was related to the induction of malignant cancer.^18^ However, the existing materials are mainly based on unswitchable structures.^19,20^ Even though there have been some magnetic^21^ or optical^22^ approaches developed to control receptor tension, the tools for tunable and reversible control of cell surface receptor clustering and thereby the modulation of cell functions with external stimuli are still very limited.

DNA has emerged as a powerful material in nanotechnology due to its ability to form programmable structures through sequence-directed hybridization.^23–25^ Moreover, DNA can be utilized to construct structure-switchable nanomachines that respond to external stimuli such as temperature, light, pH and small molecules.^26–29^ For instance, an artificial DNA nano-spring powered by protons has been constructed by assembling multiple oligonucleotides and used for controlling the distance between gold nanoparticles.^30,31^ However, the driving force of the developed DNA nano-spring is protons, which severely hindered its application for cell culture where a neutral pH is needed. On the other hand, the utilization of DNA nanostructures for controlling cellular functions has also been demonstrated.^32^ For example, a DNA origami based nanocaliper has been utilized to control the nanoscale distribution of cell surface proteins (ephrin-A5) and regulate the invasive properties of breast cancer cells.^32^ However, the DNA nanocaliper is neither structure-switchable nor multivalent and the structure design is complex.

**Scheme 1:**
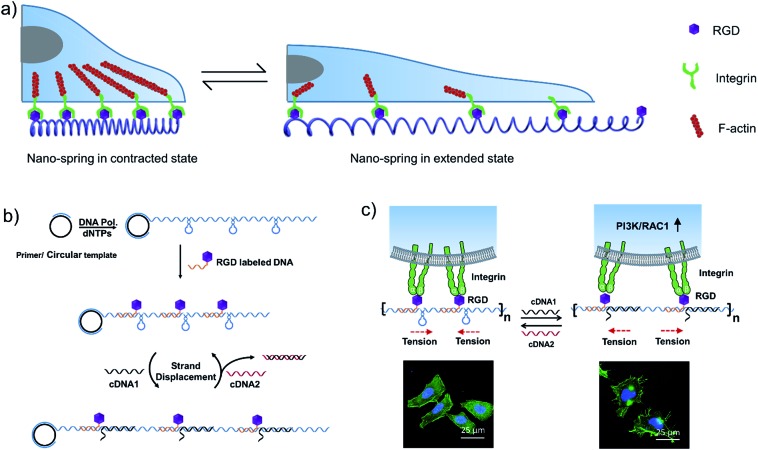
A DNA nano-spring for the reversible control of cell membrane receptor function. (a) Schematic diagram of the DNA nano-spring for the reversible control of integrin clustering. (b) The long DNA sequence, generated by rolling circle amplification, is used as a scaffold to assemble RGD (Arg-Gly-Asp) sequences in a designed form. External DNA sequences (cDNA1 and cDNA2) can be added to reversibly change the distance between the RGD sequences by strand displacement reactions. (c) DNA nano-spring triggered integrin clustering and de-clustering are able to regulate intracellular signalling pathways and the cell morphology. In the contracted state, the integrins are clustered to trigger the cells to form regular focal adhesions on the substrate. When cDNA1 is added to open the hairpin structure, the distance between the integrins increases, leading to the activation of PI3K/Rac1 signalling and changing the cell morphology from the normal morphology to having numerous cell protrusions. Cell nuclei are in blue (DAPI staining) and F-actin is in green (FITC labelled phalloidin staining). This process can be reversibly triggered by adding cDNA1 and cDNA2.

Inspired by the tentacles of marine creatures which contain multiple adhesive domains,^33^ we hypothesized that a long DNA sequence (prepared by rolling circle amplification (RCA)^34^) encoding multiple hairpin structures would be able to perform a spring-like motion with the DNA strand displacement reaction. Moreover, with the cell binding ligand (RGD) modification, the multivalent conjugates could be applied for the reversible regulation of cell membrane receptor clustering (Scheme 1a). By comparison with a previous RCA based nanomaterial,^33^ we utilized the reversible movement property of the DNA nanostructure to achieve the spring-like motion. By binding to the cell surface, the formation and deformation of the hairpin structures on the long DNA scaffold are supposed to drive the receptors to aggregate or separate, which could further affect cell membrane receptor function.

## Results and discussion

### A DNA nano-spring for the reversible control of cell receptor function

In this work, we have demonstrated that by controlling the movement of the DNA nanostructure we can induce filopodia protrusion development in Hela cells and regulate the mRNA expression of integrin related genes. The mechanism of the DNA nano-spring is shown in Scheme 1b. The long DNA scaffold was prepared by rolling circle amplification (RCA), where Phi29 DNA polymerase extended the primer along a circular template hundreds of times to yield a long single-stranded DNA. Thus, the RCA product contained hundreds of repetitive sequence units that were complementary to the circular DNA template and therefore could be pre-designed with various structures. We encoded a hairpin structure in the circular template to mimic the spring structure. The RGD (Arg-Gly-Asp) labelled DNA was hybridized with the long DNA scaffold to make multivalent ligands. With the addition of cDNA1, the hairpin structure on the scaffold could be opened and hybridized with cDNA1 to form a relatively rigid dsDNA sequence, leading to an increase in the distance between the RGD sequences. By adding cDNA2 to replace cDNA1, the distance between the RGD sequences could be decreased due to the re-formation of the hairpin structure (the strand displacement mechanism is explained by the Δ*G* calculation in the Experimental section). Notably, this process could be reversibly triggered by adding cDNA1 and cDNA2. When binding to the cell surface (Scheme 1c), the DNA nano-spring triggered integrin clustering and de-clustering controlled the cell surface receptor function and regulated the integrin related signalling pathway. Specifically, when the DNA nano-spring was in the contracted state, the integrins were supposed to cluster and the cells could form regular focal adhesions on the substrate. When cDNA1 was added to the open the hairpin structure, the distance between the integrins would increase, leading to activation of PI3K/Rac1 signalling and changing the cell morphology from the normal morphology to having numerous cell protrusions.

### Synthesis and characterization of the DNA nano-spring

**Fig. 1:**
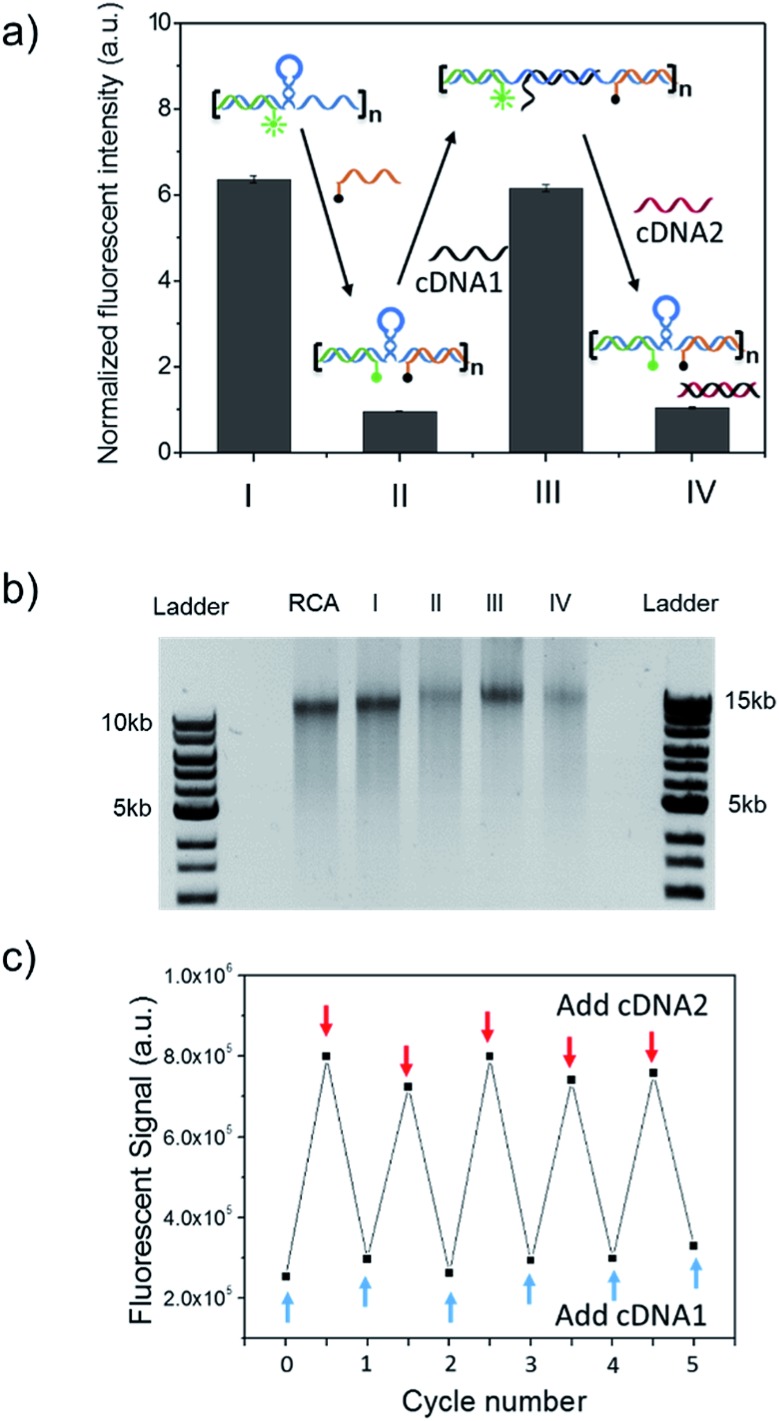
RCA products encoding multiple hairpin structures could perform a spring-like motion with a strand displacement reaction. (a) Fluorescence resonance energy transfer (FRET) measurement of the structural change of the DNA nano-spring between the extended and contracted states. (I) RCA/F-S2, (II) RCA/F-S2/Q-S1, (III) RCA/F-S2/Q-S1/cDNA1 and (IV) RCA/F-S2/Q-S1/cDNA1/cDNA2. (b) Gel electrophoresis analysis of the assembly process of the DNA nano-spring. (c) Cyclic activation of the DNA nano-spring between the extended and contracted states by adding cDNA1 and cDNA2.

The long DNA scaffold was prepared by RCA and characterized by gel electrophoresis (Fig. S1†). The length of the RCA product could be easily tuned by changing the RCA reaction time. Since a 10 min reaction produced enough RCA products and the size was more uniform than with the 30 min reaction, the 10 min reaction RCA product was used for the following experiments. To investigate whether the RCA product was capable of performing a spring-like motion, we first conducted fluorescence resonance energy transfer (FRET) analysis to monitor the structural change of the RCA product (Fig. 1a). Specifically, the long DNA sequence was consecutively hybridized with an FITC labelled oligonucleotide (F-S2, fluorophore), a dabcyl labelled oligonucleotide (Q-S1, quencher), cDNA1 and cDNA2. The distance between the dyes was expected to increase after the hybridization with cDNA1 (extended state) and decrease after strand displacement with cDNA2 (contracted state). As shown in Fig. 1a, in the contracted state (sample II) the fluorescence signal was of low intensity because of the quenching effect. While in the extended state (sample III) the fluorophore (F-S2) was well separated from the quencher (Q-S1) and the energy transfer efficiency was low, leading to a 5-fold increase of the fluorescence intensity. Addition of cDNA2 into the mixture would initiate another strand displacement and lead to the re-formation of the hairpin which quenched the fluorescence (sample IV). The DNA nano-spring assembly process was further confirmed by gel electrophoresis. As shown in Fig. 1b, the RCA product had a length of 15 kb. Along with the assembly of the nano-spring, the samples resulted in a slower and slower gel-shift mobility, corresponding to the different states of the nano-spring. Moreover, this DNA nano-spring structure could continuously cycle between the two states and the cyclic activation of the DNA nano-spring is demonstrated in Fig. 1c, which shows no obvious efficiency loss after 5 full operation cycles.

**Fig. 2:**
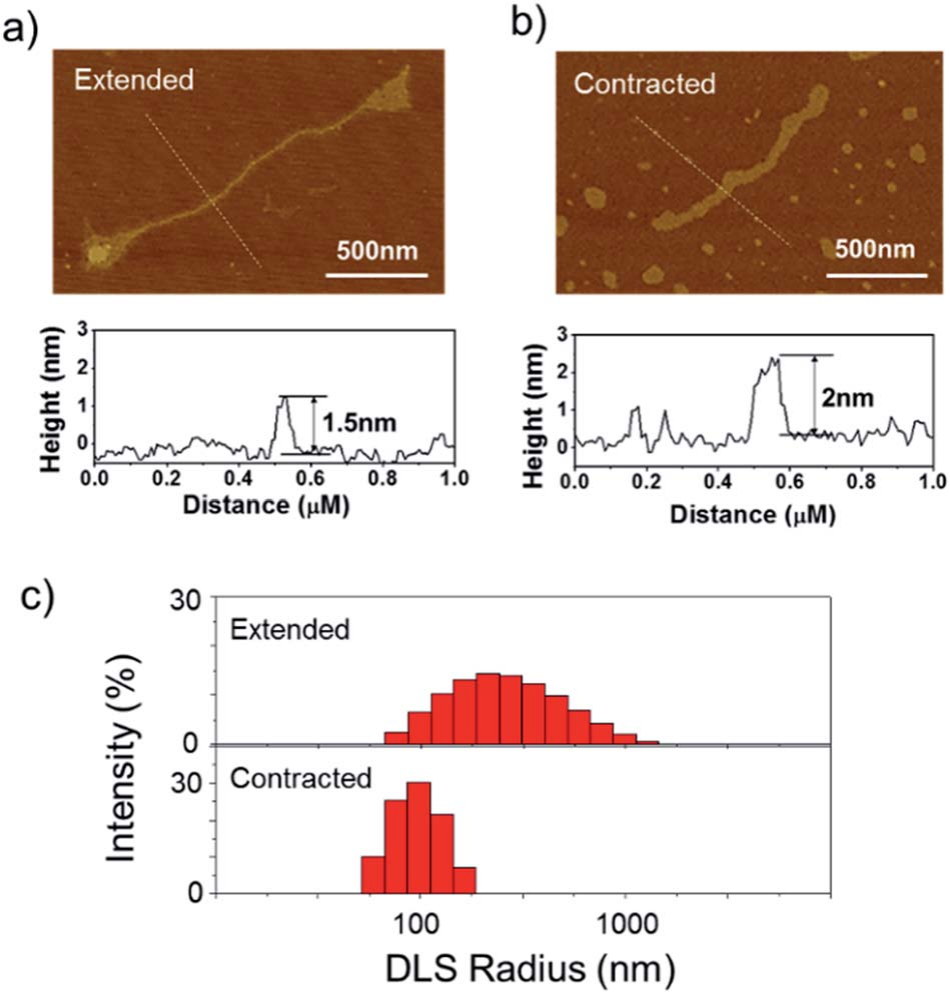
Characterization of the size change of the DNA nano-spring. Representative AFM images of the RCA products in (a) the extended state and (b) the contracted state. The height of the RCA product was measured by AFM imaging. (c) Dynamic light scattering (DLS) characterization of the RCA products in the water phase.

To further verify the spring-like motion of the RCA scaffold, we directly observed the size change of the DNA nano-spring using atomic force microscopy (AFM) and dynamic light scattering (DLS). The representative AFM images of the extended and contracted RCA products are shown in Fig. 2. The height of the RCA product in the extended state was measured to be 1.5 nm, corresponding to a dsDNA helical height.^35^ With the addition of cDNA2 to transform the RCA product into the contracted state, there was an obvious increase in the height of the RCA product (it increased to ∼2 nm). Additionally, it’s obvious that the RCA product in the contracted state was much wider than the RCA product in the extended state. However, due to the well-known AFM ‘tip broadening’ effect, the width of the RCA products cannot be accurately measured.^35^ On the other hand, the AFM images were taken under dehydration conditions which was different from the RCA product in the water phase. Therefore, we then performed DLS measurements to characterize the size change of the RCA products in the water phase. As shown in Fig. 2c, the average DLS radius of the extended and contracted samples is measured to be 312 nm and 102 nm, respectively, showing an obvious size shrinkage. The size of the RCA product detected by DLS was much smaller than that observed in the AFM images, which indicated that the RCA products may coil in the water phase. Taken together, the results collectively demonstrated that the RCA based DNA nano-spring could perform extension and contraction motions with the DNA strand displacement reaction.

### Construction of DNA nano-spring based multivalent ligands

**Fig. 3:**
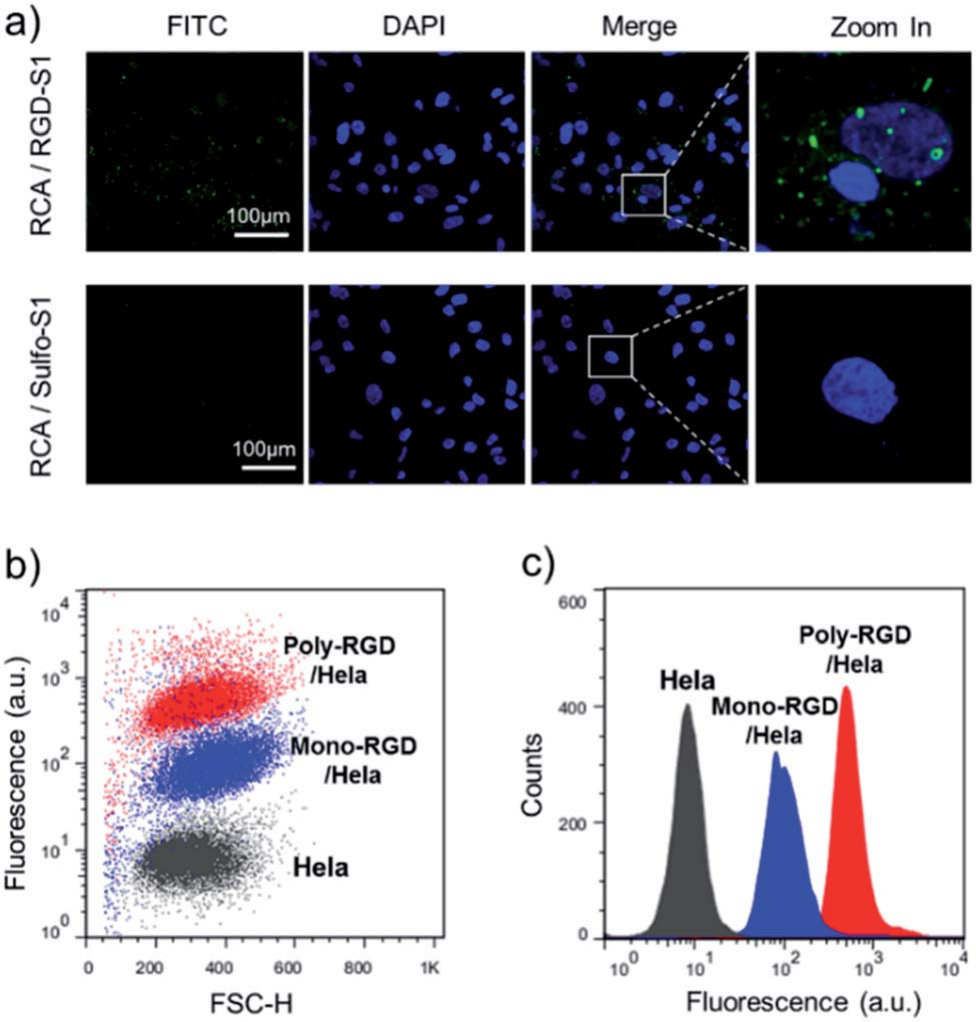
The binding affinity between poly-RGD and Hela cells. (a) Hela cells were incubated with an RGD-S1 labelled RCA scaffold or a sulfo-S1 labelled RCA scaffold. The RCA scaffolds were fluorescently labelled with F-S2, which gave an FITC fluorescence signal for imaging. (b) Flow cytometry analysis of the Hela cells binding to 1 nM FITC labelled poly-RGD (RCA : RGD = 1 : 50) or 1 nM FITC labelled mono-RGD (RCA : RGD = 1 : 1). (c) Histogram analysis of the flow cytometry data by plotting the cell count number against the detected fluorescence intensity.

The synthesized RCA scaffold was then hybridized with RGD modified oligonucleotides to construct multivalent conjugates (poly-RGD). The peptide motif RGD is an integrin-binding fibronectin fragment, usually used for interaction with integrin.^15^ The conjugation of sulfo-S1 and RGD was conducted using a heterobifunctional linker (sulfo-SMCC)^36^ and confirmed by observing an apparent shift in the molecular weights by agarose gel electrophoresis (Fig. S2†). To test the cell binding affinity of poly-RGD, we first characterized the interaction between poly-RGD and cells using confocal microscopy (Fig. 3a). As shown in Fig. 3a, poly-RGD was able to efficiently bind to Hela cells, showing bright fluorescent spots on the cell surface, while the RCA scaffold without RGD modification showed minimal non-specific cell binding. Afterwards, we further compared the cell binding affinity between poly-RGD (RCA : RGD = 1 : 50) and mono-RGD (RCA : RGD = 1 : 1) using flow cytometry analysis (Fig. 3b and c). By plotting the cell count number against the detected fluorescence intensity, we found that poly-RGD showed a significantly higher binding affinity for Hela cells than its monovalent counterpart, indicating that the multivalency of the DNA nano-spring significantly enhanced the cell binding affinity.

To test the biocompatibility of poly-RGD for cell attachment and spreading, we prepared poly-RGD coated glass for cell culture experiments. Specifically, 4 different substrates (RCA coated glass, taken as a negative control; mono-RGD coated glass; poly-RGD coated glass; and gelatin coated glass) were used in this experiment. The RCA product was stably immobilized on APTES-modified glass by electrostatic interaction. To test the stability of the RCA immobilization, we consecutively incubated the RCA coated glass with F-S2, Q-S1, cDNA1 and cDNA2 and imaged it using fluorescence microscopy. As shown in Fig. S3,† the RCA product didn’t desorb from the glass after hybridization with the complementary DNA, and the structural movement of the RCA product was not affected by the immobilization.

**Fig. 4:**
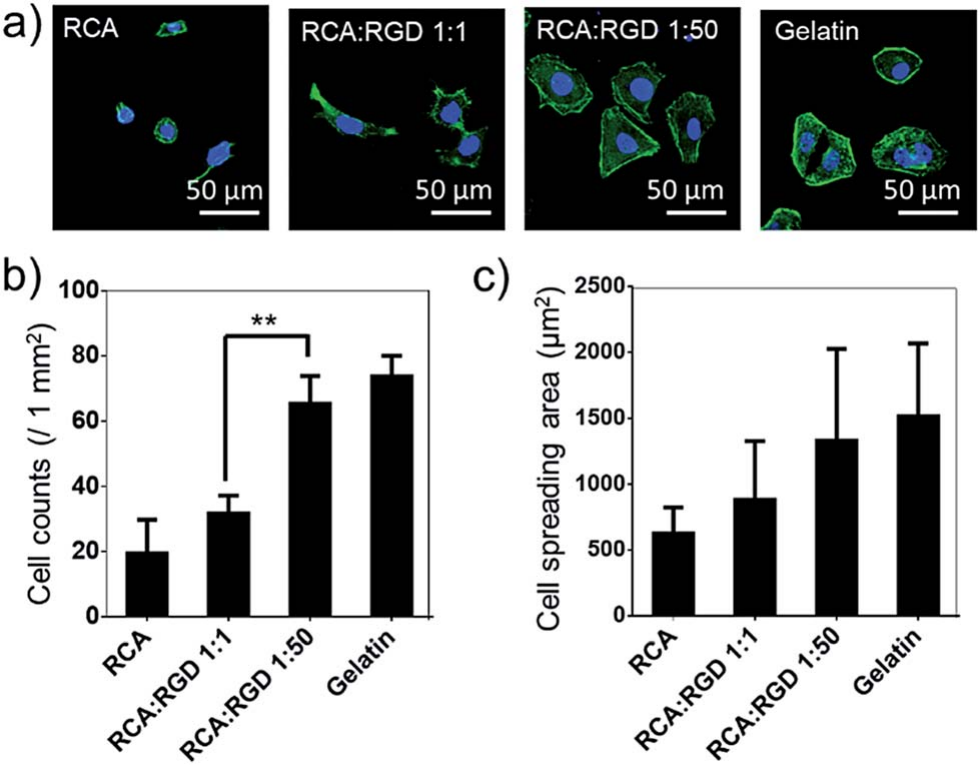
Poly-RGD coated glass for cell adhesion and spreading. (a) Fluorescence microscopy images of Hela cells cultured on 4 different substrates (the RCA product, mono-RGD, poly-RGD and gelatin) after 12 h of adhesion. Cell nuclei are in blue (DAPI staining) and F-actin is in green (FITC labelled phalloidin staining). Scale bar: 50 μm. (b) Cell density after 12 h of adhesion on the 4 different substrates as indicated. (c) Quantification of the average cell spreading area of the Hela cells cultured on the 4 different substrates as indicated. The cell spreading areas are quantified using ImageJ manually (100 cells were analyzed in each sample). Data are represented as the mean ± s.d. **P < 0.01, two-tailed Student’s *t*-test.

The poly-RGD coated glass was then applied for Hela cell culture (Fig. 4). The molar ratio of the RCA product and RGD-S1 was set to 1 : 1 and 1 : 50, respectively, for comparison. After 12 h of cell attachment, we fixed the cells and monitored the cell morphology using fluorescence microscopy and quantified the cell adhesion density and the cell spreading area. As shown in Fig. 4a, the cells cultured on poly-RGD present a similar morphology to the cells cultured on gelatin (regular cell attachment control). However, the cells cultured on the RCA product and the mono-RGD coated glass could not attach and spread well. The quantification data of the cell adhesion density and the cell spreading area also show an obvious difference between poly-RGD (1 : 50) and mono-RGD (1 : 1) (Fig. 4b and c), demonstrating that the RGD density and multivalency significantly affect cell attachment and spreading.

### Reversible control of cell membrane receptor function using the DNA nano-spring

The feasibility of the DNA nano-spring for reversibly regulating cell membrane receptor function was then explored. As demonstrated in the previous experiments (Fig. 1 and 2), the formation and de-formation of each hairpin structure on the DNA scaffold were supposed to induce nanoscale alteration of the RGD distribution. Since each of the repeated units in the RCA scaffold was 75 nt (Table S1†), the theoretical lengths of each unit in the extended and contracted states were 22.5 nm and 13.2 nm, respectively. Therefore, we could adjust the ratio of the RCA scaffold and RGD to tune the average distance between each RGD molecule. Since the RCA product was too large, its molecular weight cannot be accurately measured by gel electrophoresis. To solve this issue, we applied a fluorescence assay to estimate the length of the RCA scaffold. Specifically, 1 nM of the RCA scaffold was incubated with an F-S2/Q-S1 mix at a range of concentrations and the fluorescence signals were measured by a plate reader to draw a curve (Fig. S4†). By analyzing the inflection point of the fluorescence curve, we estimated that the length of the RCA scaffold was around 200 repeat units (15 kb). Given that the activation distance of integrin clustering was around 70 nm,^9,37,38^ we adjusted the molar ratio of the RCA product and RGD-S1 to 1 : 50 and the calculated distance between each RGD molecule was 90 nm in the extended state and 52.8 nm in the contracted state. However, it’s important to realize that under real conditions the RCA scaffold may coil (Fig. 2) and the RGD molecules won’t be equally distributed on the RCA scaffold. Thus, this theoretical calculation can only be a rough estimation. Nevertheless, since we have observed an obvious size change of the RCA scaffold in the previous experiments (Fig. 1 and 2), we hypothesize that the movement of the DNA nanostructure would be sufficient to modulate the cell behavior.

**Fig. 5:**
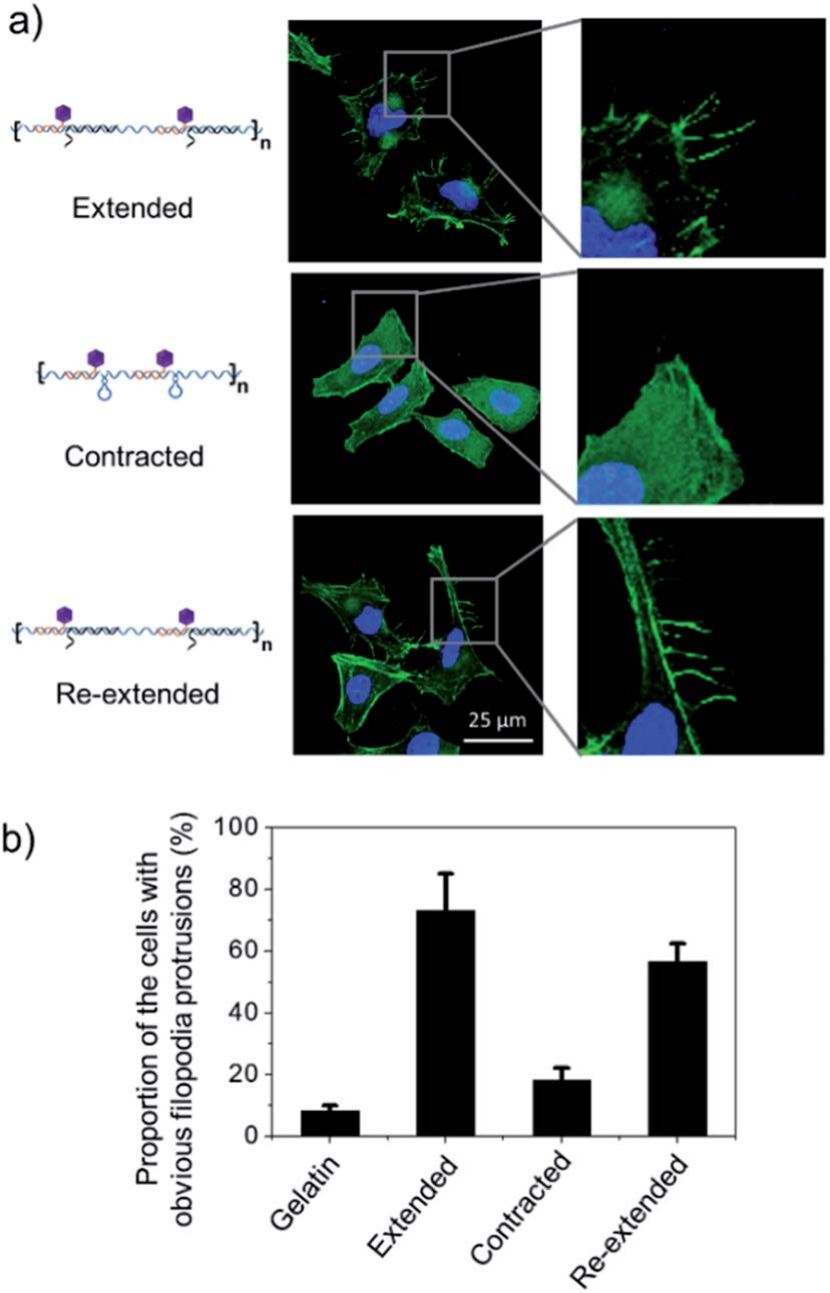
DNA nano-spring multivalent ligands regulate filopodia protrusion development of Hela cells. (a) Representative images of Hela cells cultured on 3 different substrates (extended poly-RGD, contracted poly-RGD and re-extended poly-RGD) after 12 h of cell culture. Cell nuclei are in blue (DAPI staining) and F-actin is in green (FITC labelled phalloidin staining). Scale bar: 25 μm. (b) The proportion of cells with obvious filopodia protrusions (cells cultured on 4 different substrates as indicated). Gelatin coated glass was used as a positive cell attachment control. 50 cells were analyzed for each sample. Error bars are based on 3 independent experiments.

To test this hypothesis, we cultured Hela cells on the poly-RGD coated substrate, followed by the addition of cDNA1 or cDNA2 to trigger the structural switch and monitored the variation of cell morphology and mRNA expression. As shown in Fig. 5a, when the cells are cultured on the substrate coated with poly-RGD in an extended state, many filopodia protrusions are observed at the edge of the cells, which is a typical phenotype of malignant cancer cells and may play an essential role in cell migration.^22^ After addition of cDNA2 to make the nano-spring form the contracted state, the cells tended to have a smooth surface and almost no filopodia protrusions were observed. When cDNA1 was added again to change the DNA nano-spring to a re-extended state, the filopodia protrusions were induced again. The proportion of cells with obvious filopodia protrusions (cultured on each of the 4 different substrates) was quantified by counting the number of cells manually (Fig. 5b). According to the quantification data, it’s obvious that the movement of the DNA nano-spring significantly affected cell protrusion development. A similar RGD spacing alteration induced cell morphology change was also observed in other literature reports.^39^ For example, it has been demonstrated that when the lateral spacing of RGD sequences increased, the cell morphology changed from the normal morphology to having numerous cell protrusions.^40^ Moreover, it has been demonstrated that a spacing of <70 nm between two neighboring RGD ligands would result in effective integrin clustering and focal adhesion complex formation, followed by formation of the F-actin cytoskeletal network.^7,9,39^ In agreement with the previous reports, despite the morphological changes, the cells cultured on the DNA nano-spring in the contracted state showed thicker F-actin bundles than the cells on the extended and re-extended samples (Fig. 5a), indicating the polymerization of the F-actin network.

**Fig. 6:**
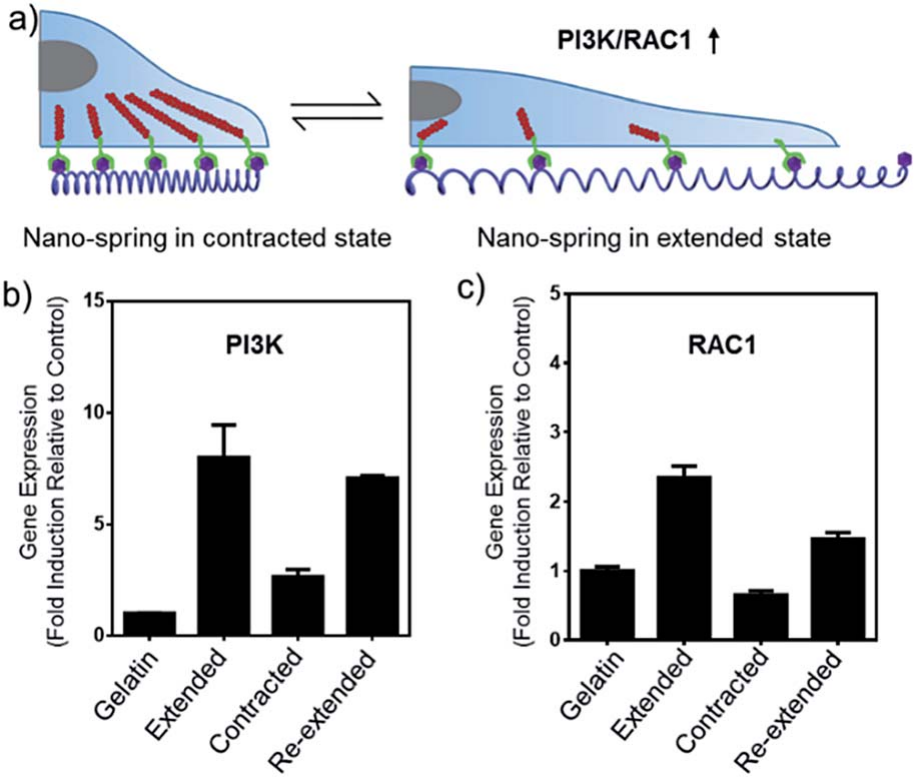
DNA nano-spring multivalent ligands are able to regulate PI3K/Rac1 mRNA expression in Hela cells. (a) Schematic diagram of the PI3K/Rac1 mRNA up-regulation induced by the movement of the DNA nano-spring. (b and c) PI3K and Rac1 expression in Hela cells cultured on 4 different substrates as indicated. The mRNA expression levels were analyzed by RT-qPCR. Gene expression from different samples was normalized to GAPDH. Error bars are based on 3 independent experiments.

The alteration of mRNA expression levels induced by the movement of the DNA nano-spring was then explored. An RT-qPCR screen of 8 genes (associated with integrin clustering) revealed a significantly altered expression in 2 of them (Table S2†). As shown in Fig. 6b and c, the PI3K/Rac1 expression, which is known to drive cell proliferation and migration,^18^ was significantly up-regulated in the cells cultured on poly-RGD in the extended state and the re-extended state. Since both PI3K and Rac1 mRNA are thought to play essential roles in cell migration,^41^ we hypothesized that the de-clustering of integrins might make the cell unable to attach well on the extracellular matrix and seek to migrate, which could be a reason for tumor metastasis. On the other hand, there is increasing evidence showing that integrin signalling associated with receptor tyrosine kinases enables cancer cells to detach from neighboring cells, migrate, and survive, which leads to tumor invasion and metastasis.^42^ For example, the Mooney group has demonstrated that reduced clustering of integrins would stop integrins from associating to form hemidesmosomes and leave the cytoplasmic tails of the integrins unbound. Therefore, multiple unbound sites are available for phosphorylation by receptor tyrosine kinases and the phosphorylation of distinct sites on the cytoplasmic tails of integrins can lead to activation of the PI3K pathway and Rac1 signalling.^2^

## Conclusions

In summary, we developed a multivalent DNA nano-spring material to reversibly control cell membrane receptor function in a biomimetic fashion. This multivalent ligand DNA nano-spring approach presents some advanced features. (1) The RCA based DNA nano-spring could perform extension and contraction motions with the strand displacement reaction. (2) The multivalency of the DNA nano-spring significantly enhanced the cell binding affinity and the receptor clustering effect. (3) The DNA nano-spring can be easily synthesized by rolling circle amplification and tuned by changing the circular template. (4) The DNA nano-spring multivalent ligands have been successfully applied for regulating mRNA expression in Hela cells and inducing cell protrusion development. Considering that the DNA structural switch can be powered by many other stimuli (pH, light, *etc.*), this DNA nano-spring approach represents a new avenue for reversibly manipulating cell surface receptor functions using a DNA nanostructure.

## Experimental section

### Materials and apparatus

All synthetic oligonucleotides (Tables S1 and S2†) were purchased from Shanghai Sangon Biological Engineering Technology & Services Co., Ltd (Shanghai, China). The dNTP mix was purchased from NEB (New England Biolabs, Ipswich, MA, USA). T4 DNA ligase, T4 PNK and Phi29 DNA polymerase were purchased from Life Technologies (Carlsbad, CA, USA). RGD (Arg-Gly-Asp) was purchased from Abcam (Cambridge, MA, USA). Sulfo-SMCC (sulfosuccinimidyl-4-(*N*-maleimidomethyl)cyclohexane-1-carboxylate) was purchased from AAT Bioquest (Sunnyvale, CA, USA). TCEP (trichloroethyl phosphate) was purchased from YEASEN (Shanghai, China). FITC labelled phalloidin was purchased from Sigma-Aldrich (St. Louis, MO, USA). A Leica TCS SP5 laser scanning confocal microscope was used for fluorescence imaging. An EnVision Multimode Plate Reader (PerkinElmer) was used for measuring the fluorescence signal. A CFX 96 real-time PCR detection system (Bio-Rad) was used for controlling the reaction temperature for rolling circle amplification and for measuring the real-time fluorescence signal of the real-time quantitative PCR for mRNA detection. A NanoDrop 2000 UV-Vis Spectrophotometer was used for measuring the nucleic acid concentration. A Wyatt DynaPro NanoStar was used for DLS measurement. A BD FACS Calibur was used for cell flow cytometry analysis.

### DNA nano-spring synthesis

The DNA nano-spring scaffold was synthesized by rolling circle amplification (RCA).^43,44^ Briefly, 5 picomoles of the circular template and primer were annealed in an RCA reaction buffer by heating up to 90 °C for 3 min and then cooled down for 15 min to room temperature. dNTPs (1 μL of 10 mM) and Phi29 DNA polymerase (1 μL of 10 U μL^–1^) were then added to the mixture, resulting in a total volume of 25 μL. The RCA reaction was performed at 37 °C for various times ranging from 1 min to 30 min. The size of the products was characterized by 1% agarose gel electrophoresis.

### The Δ*G* calculation to illustrate the strand displacement mechanism

The standard free energy change (Δ*G*) of the structures was calculated to explain the mechanism of the strand displacement reactions which drive the DNA nano-spring motion. A lower Δ*G* value suggests a more stable structure which could replace the structure with a higher Δ*G*. The Δ*G* value of the stem-loop structure was –10.37 kcal mol^–1^ whereas that Δ*G* value of the DNA scaffold and cDNA1 was –45.11 kcal mol^–1^ and the Δ*G* of cDNA1 and cDNA2 was –66.91 kcal mol^–1^. These data suggested that cDNA1 was capable of opening the hairpin structure to form a structure with a higher thermodynamic stability to make an extension motion and that cDNA2 had the capability to replace cDNA1 to make a compression motion. The Δ*G* values of the different states of the DNA nano-spring were calculated using an IDT OligoAnalyzer 3.1 (; https://www.Idtdna.com/calc/analyzer) under the conditions of 5 mM Mg^2+^ and 50 mM Na^+^.

### The bio-conjugation reaction

RGD was conjugated to sulfo-S1 DNA by the sulfo-SMCC reaction. Briefly, 2 μL of a 1 M sodium phosphate buffer at pH 5.5 and 2 μL of 30 mM TCEP in Millipore water were added to 46 μL of 1 mM thiol–DNA in Millipore water. After gently mixing, the mixture was kept at room temperature for 1 h and then purified using an Amicon-10K. 50 μL of 10 mM RGD was mixed with 1 mg of sulfo-SMCC. After a 5 min vortex, the solution was placed on a shaker for 1 h at room temperature. The mixture was then centrifuged and the insoluble sulfo-SMCC was removed. The clear solution of sulfo-SMCC-activated RGD was then mixed with the thiol–DNA solution. The resulting solution was kept at room temperature for 48 h. To remove un-reacted RGD, the solution was purified using an Amicon-10K.

### Atomic force microscopy (AFM) imaging

Freshly cleaved mica was used as an AFM substrate. The RCA product solution was diluted 10 times to make the Mg^2+^ concentration 1 mM. 10 μL of the diluted RCA product was applied to the mica for 20 min, rinsed with deionized H_2_O 15 times and dried with N_2_ gas. The samples were imaged in tapping mode using a Bruker Dimension Icon AFM.

### Substrate modification for the poly-RGD coating

The glass slides were first modified with APTES to create a positive charge on the surface. Since DNA has a negative charge, the RCA product could be strongly attached to the glass slides. Specifically, the glass slides were washed with water, ethanol and water, each for 10 min with sonication. The glass slides were then immersed in piranha solution for 1 h, followed by washing with water, ethanol and acetone. After the washing, the glass slides were incubated in 2% APTES (acetone solution) for 3 min and then washed with acetone 3 times. Finally, they were dried in an oven at 120 °C for 2 h and stored for future use. 1 mM salmon sperm DNA was used for blocking after the poly-RGD coating.

### Cell culture on the poly-RGD coated glass

Hela cells were cultured in DMEM cell culture media supplemented with 10% fetal bovine serum and 1% penicillin/streptomycin before use.

For the experiment, cells were seeded on 4 substrates (sample 1 on gelatin coated glass and samples 2, 3, and 4 on extended poly-RGD coated glass). Poly-RGD was first hybridized with cDNA1 to maintain the extended state. After 3 h of cell culture, cDNA2 was added to samples 3 and 4 to make the nano-spring structure change to a contracted state. Thus, the distance between the integrins became smaller which led to integrin clustering. After another 3 h, for sample 4, the cell culture medium was replaced and new cDNA1 was added to make the DNA nano-spring form a re-extended state, in which the formation of the rigid DNA duplex led to separation of the integrins.

After 12 h of culture, the cells were washed with PBS and fixed with paraformaldehyde. After staining with DAPI and FITC labelled phalloidin, the cells were imaged using a Leica TCS SP5 laser scanning confocal microscope.

### mRNA expression analysis

RT-qPCR was performed for mRNA expression analysis. Briefly, the total mRNA was extracted from cell samples using Trizol according to the manufacturer’s protocol. Reverse transcription was performed using reverse transcriptase and a polyT primer. For the real time PCR reaction, a 30 μL reaction mixture consisting of 15 μL 2× PCR supermix, 1.5 μL 20× primers (listed in Table S2†), 0.6 μL 50× ROX reference dye, 3 μL reverse transcription product and 10 μL H_2_O was prepared for triplicate experiments. The mRNA expression level was normalized by GAPDH.

## Conflicts of interest

There are no conflicts to declare.
