# Supplementary material for: Reversible control of cell membrane receptor function using DNA nano-spring multivalent ligands
Source: Chem Sci. 2017 Aug 18;8(10):7098–105. doi: 10.1039/c7sc02489d (PMC5637461; doi:10.1039/c7sc02489d)
Supplement: Supplementary file 1 [file SC-008-C7SC02489D-s001.pdf]

Electronic Supplementary Material (ESI) for Chemical Science.
This journal is © The Royal Society of Chemistry 2017

Supporting Information

Reversible Control of Cell Membrane Receptor Function using DNA Nano-Spring Multivalent Ligands

*Kaixiang Zhang, Ruijie Deng, Yupeng Sun, Ling Zhang, Jinghong Li**

Department of Chemistry, Key Laboratory of Bioorganic Phosphorus Chemistry & Chemical Biology, Tsinghua University, Beijing 100084, China.

*Corresponding author: jhli@mail.tsinghua.edu.cn.

List of Contents:

1. Table S1. Oligonucleotides used in this work s2
2. Table S2. Primers used for RT-PCR..s2
3. Figure S1. Gel electrophoresis analysis of the RCA product s3
4. Figure S2. Synthesis of RGD-DNA conjugate s3
5. Figure S3. The cyclic activation of the DNA nano-spring coated on glass s4
6. Figure S4. Estimation of the RCA scaffold length s4
7. Figure S5. mRNA expression of Hela cells cultured on DNA nano-spring s5

Table S1. Oligonucleotides used in this work

Name	Sequence
Circle	5'- P -GCT GGG ATA CGT GGC GCT CTT AAA GTA CTC GCG AAA AAA AAA CGC GAG TAC AAA CGC ATC TGT ACT GTA TTT CAC -3'
Ligation template	5'- ACG TAT CCC AGC GTG AAA TAC AGT -3'
cDNA1	5'- GGA GCA TGC T ACG AAA AAA AAA CGC GAG TAC AAA -3'
cDNA2	5'- TTT GTA CTC GCG TTT TTT TTT CGT AGC ATG CTC C -3'
Q-S1	5'- Dabcyl - CGC ATC TGT ACT GTA TTT CAC -3'
F-S2	5'- GCT GGG ATA CGT GGC GCT CTT - FAM -3'
Sulfo-S1	5'- SH - CGC ATC TGT ACT GTA TTT CAC -3'
RGD-S1	5'- RGD - CGC ATC TGT ACT GTA TTT CAC -3'

Table S2. Primers used for RT-PCR

Gene	Forward Primer	Reverse Primer
GAPDH	5'-CCA CTC CTC CAC CTT TGA C-3'	5'-ACC CTG TTG CTG TAG CCA-3'
FAK	5'-TCCCTATGGTGAAGGAAGT-3'	5'-TTCTGTGCCATCTCAATCT-3'
Rac1	5'-ATG CAG GCC ATC AAG TGT GTG GTG-3'	5'-TTA CAA CAG CAG GCA TTT TCT CTT CC-3'
PI3K	5'- CACGAGATCCTCTCTCTGAAATC -3'	5'- GGTAGAATTTCGGGGATAGTTACA -3'
β-actin	5'-TTGTTACAGGAAGTCCCTTGCC-3'	5'-ATGCTATCACCTCCCCTGTGTG-3'
ESR1	5'-GCCAAATTGTGTTTGATGGATTAA-3'	5'-GACAAAACCGAGTCACATCAGTAATAG-3'
ANKRD	5'-AGTAGAGGAACTGGTCACTGG-3'	5'-TGGGCTAGAAGTGTCTTCAGAT-3'
CTGF	5'-AGGAGTGGGTGTGTGACGA-3'	5'-CCAGGCAGTTGGCTCTAATC-3'
LATS1	5'-CTCTGCACTGGCTTCAGATG-3'	5'-TCCGCTCTAATGGCTTCAGT-3'

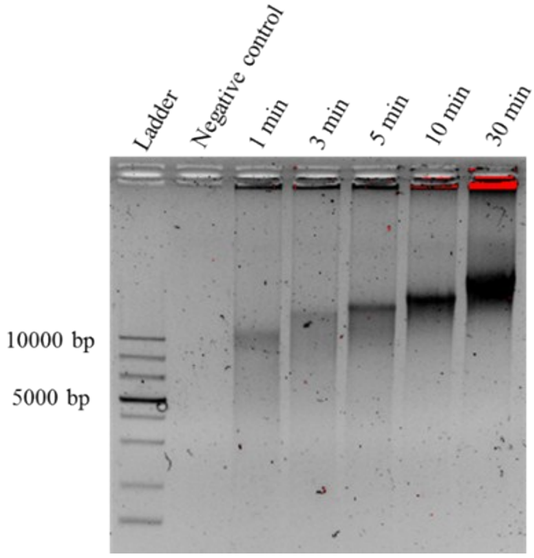

Figure S1. Gel electrophoresis analysis of the RCA product at different reaction time. The size of RCA product could be tuned by controlling the reaction time. The RCA product is a long single strand DNA.

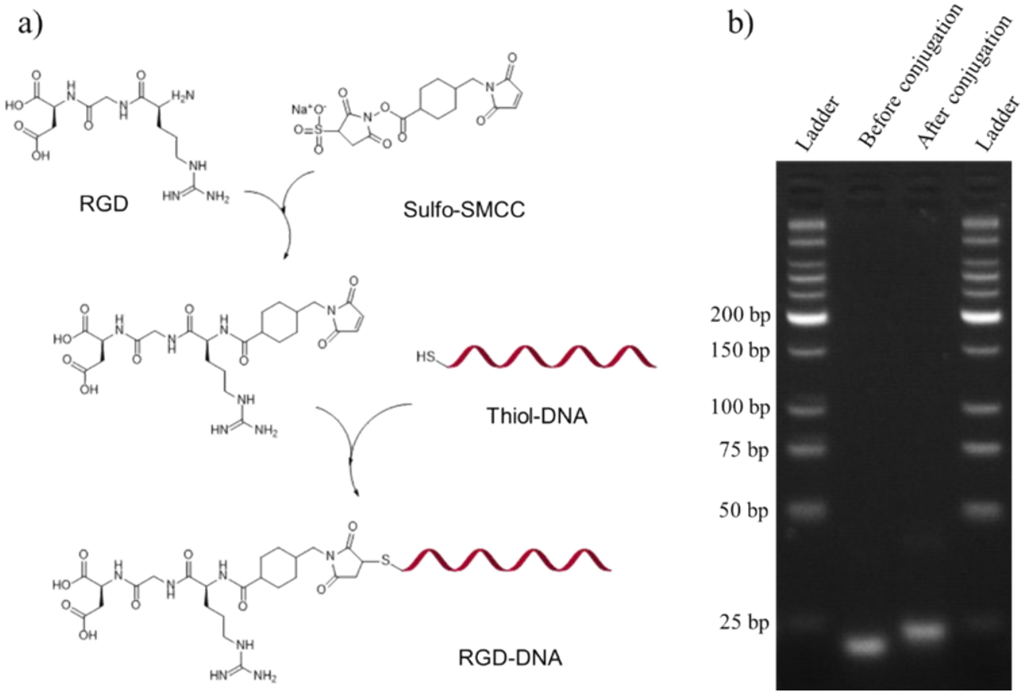

Figure S2. Synthesis of RGD-DNA conjugate. a) Synthetic pathway for RGD and oligonucleotide conjugation. b) Agarose gel electrophoresis characterization of the RGD-DNA conjugates.

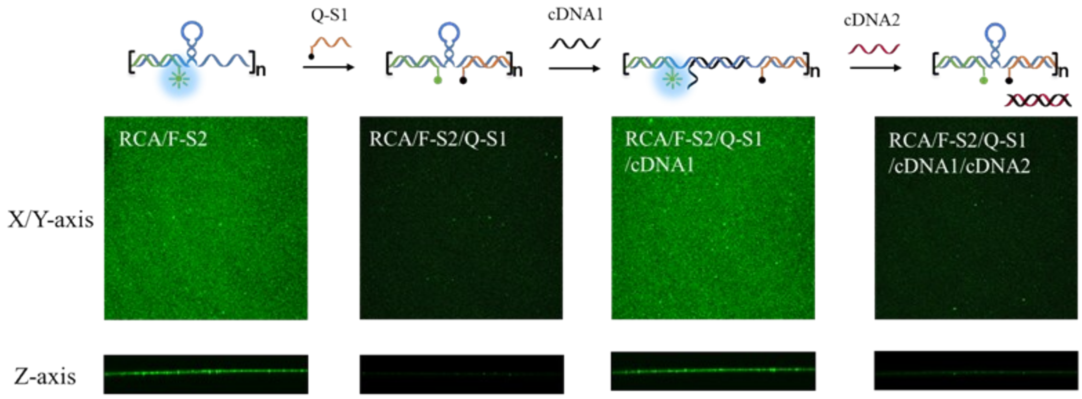

Figure S3. Fluorescent microscopic images of the cyclic activation of the DNA nano-spring coated on glass substrate. 1 nM DNA nano-spring was incubated with APTES modified glass for 30 mins and then washed with H_2O. The DNA nano-spring coated glass was then consecutively hybridized with F-S2, Q-S1, cDNA1 and cDNA2 .

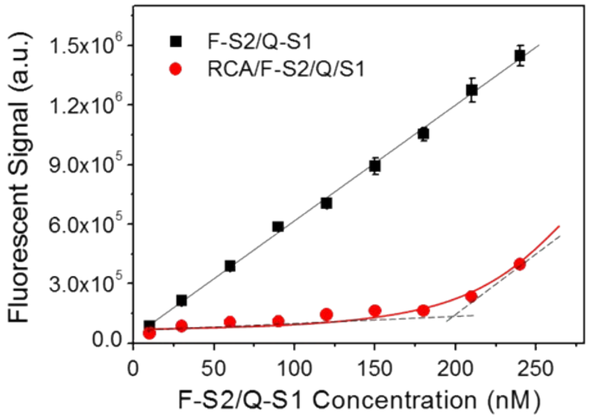

Figure S4. Fluorescent measurement to estimate the length of RCA scaffold. 1 nM RCA scaffold was hybridized with different concentration of F-S2/Q-S1 mix (0 nM – 250 nM). When F-S2 and Q-S1 were binding to the RCA scaffold, the fluorescent signal was quenched. Along with the increase of F-S2/ Q-S1 concentration, the RCA scaffold would be saturated at a specific concentration and the fluorescent signal would increase linearly afterwards. Therefore, by observing the the inflection point of the fluorescent curve, the length of RCA scaffold could be estimated.

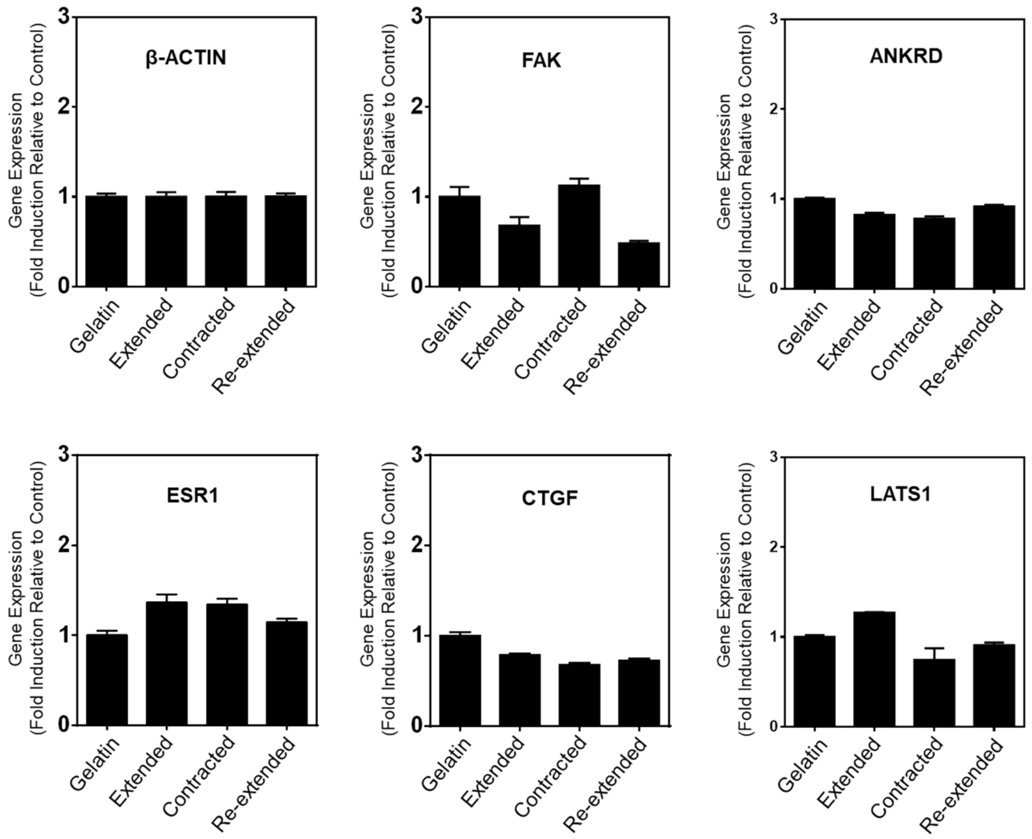

Figure S5. mRNA expression of Hela cells cultured on 4 different substrates as indicated. The mRNA expression levels were analyzed by RT-qPCR. Gene expression from different samples was normalized to GAPDH. Error bars are based on 3 independent experiments.
